# The Daily Experiences of Hispanic and Latino Dementia Caregivers: Findings From the Nuestros Días Study

**DOI:** 10.1093/geroni/igaf122.1701

**Published:** 2025-12-31

**Authors:** Frank Puga, Natashia Bibriescas, Krystal Kittle

## Abstract

Hispanic and Latino family caregivers of people living with Alzheimer’s disease and related dementias (ADRD) experience high levels of stress, which can negatively impact their mental health and caregiving behaviors. However, little is known about the day-to-day experiences that contribute to these outcomes. The Nuestros Días (Our Days) study, a fully remote daily diary observational cohort study, addresses this gap by examining daily stress, mood, and well-being among Hispanic and Latino ADRD caregivers. This symposium presents an overview of the Nuestros Días Study, key findings, and implications for developing culturally responsive interventions for H&L caregivers. Drs. Bibriescas and Puga will provide an overview of the study’s methodology, sample characteristics, and initial findings on daily variations in caregiving-related stress and mental health. Ms. Rodriguez Colmenares will discuss how coping strategies influence daily caregiver mental health, highlighting the link between dysfunctional coping and increased depression, anxiety, and stress. Ms. Mildrum Chana will discuss the relationship between social media use and caregiver well-being, emphasizing the risks of passive social media engagement for mental health. Ms. Poe will present findings on how caregivers’ involvement in care-related decision-making influences daily anxiety and depressive symptoms. Finally, Dr. DiFiglia will present qualitative findings on caregivers’ daily challenges and the strategies they use to manage stress, offering insights into culturally relevant approaches to stress reduction. Findings from Nuestros Días provide critical insights into the daily experiences of Hispanic and Latino dementia caregivers, offering implications for interventions that enhance well-being and resilience.

